# The psychological impact of the COVID-19 pandemic and confinement period on a tunisian sample

**DOI:** 10.1192/j.eurpsy.2021.760

**Published:** 2021-08-13

**Authors:** M. Bejar, B. Ben Mohamed, N. Faouel, R. Belhadj Ali, F. Zaafrane, L. Gaha

**Affiliations:** 1 Research Laboratory, R05es10, Faculty Of Medicine Of Monastir, Tunisia, Psychiatry Department, Fattouma Bourguiba University Hospital, Monastir, Tunisia, monastir, Tunisia; 2 Psychiatry, Faculty of medicine of Monastir, Monastir, Tunisia

**Keywords:** COVID19, eating disorders, Depression, Anxiety

## Abstract

**Introduction:**

Coronavirus disease 2019, is now a global pandemic that has spread rapidly causing many deaths. Most countries have opted for compulsory confinement which had repercussions on mental health and well-being.

**Objectives:**

The purpose of this study is to assess mental health consequences during the confinement period.

**Methods:**

This is a cross-sectional descriptive study of 360 Tunisians in April and May 2020. We used an anonymous E-questionnaire that included a socio-demographic fact sheet, The HAD questionnaire, and a Q-EDD questionnaire to explore eating disorders.

**Results:**

The subjects of our sample were mostly males with a mean age of 31. The body mass index was 25.5 (range 16.10 -46.24), 15% suffered from obesity. Half of the subjects were single and 6.7% spent the confinement time alone. 11.1% were smokers while 4.4% were alcohol users. The HAD-A and the HAD-D scores had an average of 9.1 and 8.48 respectively.A pathological threshold of anxiety and depression was found in 20% and 30% of the sample respectively.15% had an eating disorder: 76% had binge eating, 20% had bulimia and 17% had anorexia. In our study, we found an association between eating disorder and obesity, single marital status (p=0.007), living alone (p=0.001), history of depression (p=0.046), anxiety (p=0.049) and depression (p=0.038).

**Conclusions:**

Reduced social interactions, decreased physical activity and increased stress are potentially harmful causes for our brain. Confining the population for several weeks has a negative impact on our physical and mental health. A crisis unit has been formed in Tunisia to help subjects overcome these psychological difficulties.

